# Characterization of Maternal Psychosocial Stress During Pregnancy: The Healthy Start Study

**DOI:** 10.1089/whr.2022.0011

**Published:** 2022-08-04

**Authors:** Satvinder K. Dhaliwal, Dana Dabelea, Angela E. Lee-Winn, Deborah H. Glueck, Greta Wilkening, Wei Perng

**Affiliations:** ^1^ Lifecourse Epidemiology of Adiposity and Diabetes (LEAD) Center, University of Colorado Denver Anschutz Medical Campus, Aurora, Colorado, USA.; ^2^ Department of Epidemiology, Colorado School of Public Health, University of Colorado Denver Anschutz Medical Campus, Aurora, Colorado, USA.; ^3^ Department of Pediatrics, School of Medicine, University of Colorado Denver Anschutz Medical Campus, Aurora, Colorado, USA.; ^4^ Department or Neuropsychology, Colorado Children's Hospital, Aurora, Colorado, USA.

**Keywords:** psychosocial stress, principal component analysis, maternal stress

## Abstract

**Objective:**

To capture multidimensional maternal psychosocial stress using responses from the Edinburgh Postnatal Depression Scale (EPDS) and Cohen's Perceived Stress Scale (PSS) administered during pregnancy, and to identify sociodemographic, biological, and health behavioral correlates of the stress domains.

**Methods:**

Using data from 1,079 pregnant women, we implemented principal component analysis on EPDS and PSS responses and retained factors based on the Scree plot and Eigenvalues >1. We then used linear regression to identify perinatal correlates of each domain.

**Results:**

We identified three stress domains: “Feeling Overwhelmed,” “Anhedonia,” and “Lack of Control,” which accounted for 10.6% of variance in questionnaire responses. In multivariable analyses, household income ≤$70,000 (*β* = 0.21 confidence interval [95% CI: 0.05–0.39]), primiparity (0.36 [0.02–0.71]), inadequate (0.21 [0.04–0.39]) or excessive gestational weight gain (0.27 [0.11–0.42]), and Healthy Eating Index (HEI) score ≤57 (0.14 [0.00–0.28]) were associated with Feeling Overwhelmed. Older age (0.02 [0.00–0.03] per 1-year), Hispanic ethnicity (0.19 [0.00–0.38]), and HEI score ≤57 (0.15 [0.02–0.28]) were associated with Anhedonia. Non-Hispanic Black race/ethnicity (0.37 [0.10–0.63]), not having graduated from college (0.16 [−0.02 to 0.35]), having a partner born outside the United States (0.17 [−0.02 to 0.37]), household size of ≥5 persons (0.21 [−0.02 to 0.37]), receiving public assistance (0.18 [−0.02 to 0.37]), and prenatal smoking (0.32 [0.05–0.59]) were associated with Lack of Control.

**Conclusions:**

Three domains of maternal psychosocial stress during pregnancy (Feeling Overwhelmed, Anhedonia, and Lack of Control) were differentially related to sociodemographic, biological, and health behavioral characteristics that may be targets for interventions to ameliorate stress in pregnant women.

**Clinical Trial Registry:**

*:* The Healthy Start study is registered as an observational study at clinicaltrials.gov (NCT #002273297).

## Introduction

Psychosocial stress, stemming from experiences of bias, discrimination, and/or trauma, contribute to the origin and progression of many complex chronic diseases.^1^ Stress during pregnancy is of particular concern, given that maternal experiences and exposures during this life stage affect health not only of the mother but also has implications for health and development for her child.^2^ A growing literature implicates maternal psychosocial stress and/or depression in a range of adverse outcomes in the offspring, including low birth weight, preterm delivery, attention-deficit hyperactivity disorder, and autism spectrum disorder,^3–13^ potentially through *in utero* programming of the hypothalamic pituitary axis and/or the inflammatory response.^3,6,7,14^

To date, most studies assessed maternal psychosocial stress by self-report of traumatic life events, or implemented questionnaires such as Cohen's Perceived Stress Scale (PSS) and the Edinburgh Postnatal Depression Scale (EPDS) to query a woman's perceived psychosocial stress on a continuous scale or through established thresholds.^3^ The former approach is likely to capture more extreme adverse experiences that may not be generalizable. The latter approach assesses specific aspects of psychosocial stress across a continuous spectrum that is likely relevant to most women in a general-risk setting. For instance, the PSS captures the degree to which life circumstances are deemed overwhelming or unpredictable, whereas the EPDS captures anxious and depressive symptoms. However, single scales do not capture the multiple layers of psychosocial stress experienced simultaneously.

An alternate approach to singular assessments of psychosocial stress is to combine multiple assessments to capture the multidimensionality of psychosocial stress.^15,16^ While these approaches are less commonly used to evaluate maternal psychosocial stress during pregnancy, a recent study led by Maxson et al. used k-means clustering^17^ to categorize 1,313 pregnant women into mutually exclusive groups of distinct psychosocial stress profiles based on responses to five questionnaires. The authors identified three stress profiles characterized by varying degrees of depression, psychosocial stress, and interpersonal support.

Women in the “resilient” group exhibited low depression and perceived stress, and high interpersonal support, paternal support, and self-efficacy; the “vulnerable” group was marked by high depression and perceived stress, and low interpersonal support, paternal support, and self-efficacy; and women in the “moderate” group were between the resilient and vulnerable profiles on all domains. While such an approach offers more realism and generalizability than traumatic experiences, the mutually exclusive groups of participants do not reflect the fact that individuals experience varying degrees of stress in more than one domain.

In this study, we conduct an exploratory analysis to characterize multifaceted domains of psychosocial stress among pregnant women in the Healthy Start cohort by implementing principal component analysis (PCA) on EPDS and PSS responses. We chose PCA over other data reduction methods for ease of interpretation and realism as this procedure assigns every individual in the population a score for each latent construct, rather than forcing individuals into mutually exclusive domains.^18^ Secondarily, we sought to identify sociodemographic, biological, and health behavioral correlates of these domains during the perinatal period, which may serve as targets for prevention or mitigation of psychosocial stress. We hypothesized that we would retain at least two domains of maternal psychosocial stress and expected to observe variation in maternal psychosocial stress domain scores based on sociodemographic, biological, and behavioral characteristics.

## Methods

### Study population

The Healthy Start Study is a prospective cohort study of 1,410 pregnant women and their children recruited in Denver, CO, from 2010 to 2014.^19^ Women completed two research visits at ∼17 (“early pregnancy visit”) and 27 (“late pregnancy visit”) gestational weeks, during which we administered questionnaires inquiring on sociodemographic characteristics and health behaviors. For this study, we excluded participants who did not complete the EPDS or PSS, resulting in an analytic sample of 1,079 women. The subsample for this study was similar to the entire Healthy Start sample with respect to race/ethnicity, education level, birth country, partner's birth country, household income, parity, and smoking status (data not shown; available upon request).

### Implementation of psychiatric questionnaires (EPDS and PSS)

At the early pregnancy visit, women completed the EPDS and Cohen's PSS.^20,21^ The EPDS is a validated 10-item questionnaire used to assess risk of postpartum depression (PPD) by querying the respondent's feelings and level of satisfaction or dissatisfaction with regular activities and behaviors in the prior 7 days by a ranking scale from 0 to 3 (0 = As much as I always could, 1 = Not quite as much now, 2 = Definitely not as much now, and 3 = Not at all). The PSS is a 10-item questionnaire used to assess the degree to which life situations are deemed unpredictable, uncontrollable, and overwhelming.^20,22^ Although the EPDS is validated for assessing PPD, lower scores that do not reach the PPD diagnosis threshold (<13) can be interpreted as indications of maternal distress in clinical settings.^23^

The PSS has been shown to have high internal reliability in pregnant populations and has been validated for depressive and physical symptomatology of stress in nonpregnant populations.^24,25^ Respondents rank a series of situations, on a 5-point scale describing how often they experienced the burden of a scenario in the past month, where 0 = never, 1 = almost never, 2 = sometimes, 3 = fairly often, and 4 = very often.

### Maternal characteristics

#### Sociodemographic characteristics

At the early pregnancy visit, women provided information on sociodemographic characteristics and health behaviors. Using questionnaires, participants indicated their age in years and race/ethnicity based on the following categories: Hispanic or Latina, White or Caucasian, Black, Asian or Pacific Islander, American Indian or Alaskan Native, and other. For the analysis, racial/ethnic categories were collapsed into Hispanic, non-Hispanic White, non-Hispanic Black, and non-Hispanic other due to small cell sizes in each of the original categories.

At the early pregnancy visit, women also provided information about their education level (less than high school, high school degree or GED, some college or associate's degree, 4 years of college, and graduate degree), her own as well as her partner's country of birth (United States or non-United States), and annual household income (≤$20,000, 20,001 to <40,000, $40,000–70,000, and ≥$70,000). At the same visit, we also inquired on the number of persons living in the woman's household, and use of public assistance programs (*e.g.*, Women, Infants, and Children program or Food Stamps).

#### Biological characteristics during the perinatal period

Medical records provided information on gestational age at delivery, which we categorized as <37 versus ≥37 weeks, corresponding with preterm versus term delivery.^26^ Prepregnancy body mass index (BMI) (kg/m^2^) was calculated using maternal height measured at the first study visit and maternal weight, which was either recorded by a medical provider at the first prenatal visit (91%) or self-reported at the first research visit (9%).^27^ We then categorized BMI using standard thresholds.^28^ Gestational weight gain (GWG) was calculated as the difference between the last available weight measurement during pregnancy and prepregnancy weight.^27^ GWG measures were classified as insufficient, adequate, or excessive, as recommended by the Institute of Medicine 2009 guidelines.^29^ Parity was self-reported by women at enrollment and gestational diabetes mellitus was documented from the medical record.^30^

#### Health behaviors

Women reported their smoking habits during early pregnancy, which we categorized as ever smoked during gestation versus did not smoke during gestation. We collected dietary intake data using the multiple-pass Automated Self-Administered 24-hour Dietary Recall (ASA24) questionnaire starting in the first trimester with an average of three to four recalls per woman.^31,32^ We used these data to estimate Healthy Eating Index (HEI)—2020 score and dichotomized as ≤57 versus >57^31,32^ to indicate low versus high diet quality as was previously done. Maternal physical activity data were collected using the Pregnancy Physical Activity Questionnaire.^33,34^ We dichotomized physical activity per week at 150 minutes per week as the threshold for adequate physical activity per recommendations of the Centers for Disease Control and Prevention.^35^

### Statistical analyses

Before our main analysis, we examined the univariate and frequency distributions of key variables to identify deviations from normality and missing values. Then, we implemented the main analysis in two steps, described below.

#### Step 1: characterize domains of maternal stress

To characterize the domains of maternal stress, we first standardized participants' responses to the EPDS and PSS such that a higher value corresponds with higher stress, which entailed reverse coding PSS questions 4, 5, 7, and 8 (Supplementary Table S1).

Next, we entered the standardized questionnaire responses (20 variables) into a principal component analysis (PCA), an unsupervised dimension reduction approach that yields orthogonal latent constructs (factors) based on the correlations among the original variables, yielding distinct (*i.e.*, uncorrelated) domains of maternal stress that are naturally occurring within the study population.^18^ We used the Scree plot and standard criterion of Eigenvalues >1 to determine the number of factors to retain for subsequent analyses.^18^ For these factors, we subsequently derived a factor score, or a normally distributed variable that represents the extent to which an individual's EPDS and PSS responses reflect the psychosocial stress domain captured by that factor. Each individual was assigned a factor score for each stress domain, capturing experiences of stress in multiple domains. We interpreted the factors based on the variables with the highest factor loadings, focusing on those with factor loading >0.4.

#### Step 2: identify perinatal correlates of maternal stress domains

After deriving factor scores for the maternal stress domains of interest, we used linear regression to identify sociodemographic, biological, and health behavioral correlates of each domain during the perinatal period. In these models, each perinatal characteristic was the independent variable and the factor score for a given domain was the dependent variable. After identifying significant associations in unadjusted analyses (alpha = 0.10, given the exploratory nature of this analysis), we built a multivariable model for each stress domain.

In the multivariable models, perinatal characteristics that were significant in unadjusted analyses were considered for inclusion, while also taking into account the temporal relationship and correlation among variables (*e.g.*, if both prepregnancy BMI and GWG were significant in unadjusted analyses, we included only the former in the multivariable model since it is upstream of and directly affects the latter). For all models, we assessed jackknifed studentized residuals to confirm assumptions of normality. Data management and statistical analyses were conducted in SAS version 9.4, The SAS Institute (Carey, NC).

## Results

Table 1 shows descriptive statistics of the study population. The average age of women at enrollment was 28 years (standard deviation = 6.2). Approximately half (53.3%) identified as non-Hispanic White, 38.8% as Hispanic, 14.6% as non-Hispanic Black, 2.9% as non-Hispanic Asian, and 3.4% as another race/ethnic identity. The majority of participants (85%) and their partners (65.3%) were born in the United States. Most women (66.9%) attended college. Approximately half (51.1%) of the sample reported an annual household income ≤$40,000 and 36.8% received public assistance. Additional characteristics are in Table 1.

**Table 1.** Background Characteristics of 1,079 Healthy Start Women Included in This Report

	*N* ^a^	%
**Maternal sociodemographic characteristics**		
Age at early pregnancy interview		
16–24 years	360	33.4
25–29 years	277	25.7
30–34 years	307	28.5
35 years or older	135	12.5
Race/ethnicity		
Hispanic	278	38.8
Non-Hispanic White	575	53.3
Non-Hispanic Black or African American	158	14.6
Non-Hispanic Asian	31	2.9
Other	37	3.4
Birth country		
United States	917	85
Outside United States	162	15
Partner's birth country		
United States	704	65.3
Outside United States	172	15.9
Education		
Less than 12th grade	162	15
High school degree or GED	196	18.2
Some college or associate's degree	252	23.4
Four years of college (BA, BS)	235	21.8
Graduate degree	234	21.7
Annual household income		
$20,000 or less	354	32.8
$20,001–40,000	197	18.3
$40,001–70,000	150	13.9
$70,000	163	15.1
No. of persons in household		
1–2	437	40.5
3–4	496	46
5–6	114	10.6
7 or more	25	2.3
Receipt of public assistance		
Yes	397	36.8
No	674	62.5
**Biological characteristics**		
Prepregnancy BMI		
Underweight	34	3.2
Normal weight	529	49
Overweight	293	27.2
Obese	220	20.4
Parity (pregnancy index)		
1	1038	96.2
2	40	3.7
3	1	0.1
Gestational weight gain		
Inadequate	281	26
Adequate	305	28.3
Excessive	487	45.1
Gestational diabetes		
Yes	42	3.9
No	925	85.7
Gestational age at delivery		
<37 weeks	67	6.2
≥37 weeks	980	90.8
**Health behaviors**		
Smoked during pregnancy		
Did smoke	89	8.3
Did not smoke	990	91.8
HEI		
HEI >57	249	23.1
HEI ≤57	792	73.4
Physical activity		
≥150 min/week	644	59.7
<150 min/week	435	40.3

^a^ Frequencies and percentages may not sum to the total sample size due to missing values.

BMI, body mass index; HEI, Healthy Eating Index.

Supplementary Table S1 shows the distribution of responses to the PSS and EPDS questionnaires, along with the coding scheme to standardize the responses before PCA analysis. Supplementary Table S2 shows the correlation coefficients across sociodemographic, biological, and health behavioral characteristics, which we assessed as correlates of each psychosocial stress domain.

After implementing the PCA on the EPDS and PSS responses, we retained three factors with Eigenvalues >1, which together explained 10.6% of variance in the questionnaire responses. Based on the variables with highest factor loadings (Table 2), we refer to Domain 1 (4.7% of variance) as “Feeling Overwhelmed,” Domain 2 (3.0% of variance) as “Anhedonia,” and Domain 3 (2.9% of variance) as “Lack of Control.”

**Table 2.** Factors of Maternal Stress Retained from Principal Component Analysis

	Factor 1: Feeling Overwhelmed	Factor 2: Anhedonia	Factor 3: Lack of Control
Anxious or worried	0.70431^a^	0.07944	0.07819
Nervous or stressed	0.70386^a^	0.24744	0.14447
Upset because of unexpected occurrence	0.67618^a^	0.25203	0.18226
Scared or panicky	0.66123^a^	0.1504	0.13945
Blamed myself unnecessarily	0.63422^a^	0.08366	0.19448
Unable to control important things	0.62345^a^	0.19675	0.18511
Angered by things that are outside control	0.5662^a^	0.2645	0.30896
Things getting on top of me	0.54054^a^	0.32207	0.30904
Could not cope with responsibilities	0.53827^a^	0.28768	0.18949
Things piling up	0.53296^a^	0.36465	0.35528
Unable to look forward with enjoyment	0.06923	0.7671^a^	0.21553
Unable to laugh at things	0.11308	0.74843^a^	0.10326
So unhappy that I have difficulty sleeping	0.35273	0.62032^a^	0.13952
Sad or miserable	0.44192	0.60995^a^	0.22045
So unhappy that I have been crying	0.48246	0.52631^a^	0.21923
Lack of confidence in handling personal problems	0.12972	0.13786	0.76308^a^
Things not going your way	0.22736	0.22692	0.7418^a^
Not on top of things	0.23656	0.20647	0.73725^a^
Unable to control irritations	0.22898	0.07861	0.72429^a^

^a^ Factor loading scores >0.4.

Variance explained: 4.65% Factor 1; 2.96% Factor 2; 2.88% Factor 3.

In unadjusted analyses (Table 3), non-White race/ethnicity, lower educational attainment, lower annual household income, three or more prior births, inadequate or excessive GWG, smoked during pregnancy, and poor diet quality (HEI ≤57) were each associated with a higher score for Domain 1 (Feeling Overwhelmed). Correlates of a higher score for Domain 2 (Anhedonia) included younger age, non-White race/ethnicity, non-United States nativity, having a partner of non-United States nativity, lower educational attainment and household income, larger household size, receipt of public assistance, having prepregnancy BMI outside the normal range (18.5 ≥ BMI <25 kg/m^2^), and poor diet quality. Younger age, non-White race/ethnicity, being born outside the United States, non-United States nativity, lower educational attainment and household income, larger household size, receipt of public assistance, having prepregnancy BMI outside the normal range, smoking during pregnancy, and poor diet quality were associated with higher score for Domain 3 (Lack of Control).

**Table 3.** Bivariate Associations of Maternal Sociodemographic, Biological, and Health Behavioral Characteristics During the Perinatal Period with Maternal Stress Domains

	Domain 1: Overwhelmed		Domain 2: Anhedonia		Domain 3: Lack of control	
	Estimate (95% CI)	*p*	Estimate (95% CI)	*p*	Estimate (95% CI)	*p*
**Maternal sociodemographic characteristics**						
Age						
16–24 years	0.00 (Reference)	0.20	**0.00 (Reference)**	**0.003**	**0.00 (Reference)**	**<0.0001**
25–29 years	−0.18 (−0.33 to −0.02)		**−0.12 (−0.28 to 0.03)**		**−0.46 (−0.61 to −0.31)**	
30–34 years	−0.15 (−0.30 to 0.01)		**−0.28 (−0.44 to −0.13)**		**−0.70 (−0.84 to −0.55)**	
35 years or older	−0.07 (−0.27 to 0.12)		**−0.16 (−0.35 to 0.04)**		**−0.42 (−0.61 to −0.23)**	
Race/ethnicity						
Hispanic	**−0.02 (−0.16 to 0.13)**	**0.05**	**0.38 (0.24 to 0.53)**	**<0.0001**	**0.48 (0.34 to 0.61)**	**<0.0001**
Non-Hispanic White	**0.00 (Reference)**		**0.00 (Reference)**		**0.00 (Reference)**	
Non-Hispanic Black	**0.13 (−0.04 to 0.31)**		**0.28 (0.10 to 0.45)**		**0.64 (0.47 to 0.81)**	
Non-Hispanic Asian	**0.26 (−0.10 to 0.62)**		**0.04 (−0.31 to 0.4)**		**0.26 (−0.09 to 0.61)**	
Other	**0.40 (0.07 to 0.74)**		**0.46 (0.13 to 0.78)**		**0.37 (0.04 to 0.69)**	
Birth country						
United States	0.00 (Reference)	0.44	**0.00 (Reference)**	**<0.0001**	**0.00 (Reference)**	**0.006**
Outside United States	−0.07 (−0.23 to 0.10)		**0.37 (0.21 to 0.54)**		**0.24 (0.07 to 0.40)**	
Partner's birth country						
United States	0.00 (Reference)	0.93	**0.00 (Reference)**	**0.0006**	**0.00 (Reference)**	**<0.0001**
Outside United States	0.01 (−0.15 to 0.17)		**0.27 (0.12 to 0.42)**		**0.37 (0.21 to 0.53)**	
Education level						
Less than 12th grade	**0.13 (−0.07 to 0.33)**	**0.05**	**0.60 (0.41 to 0.80)**	**<0.0001**	**1.06 (0.87 to 1.25)**	**<0.0001**
High school degree or GED	**0.12 (−0.07 to 0.31)**		**0.44 (0.25 to 0.62)**		**0.55 (0.38 to 0.73)**	
Some college or associate's degree	**0.22 (0.05 to 0.40)**		**0.37 (0.20 to 0.54)**		**0.44 (0.28 to 0.61)**	
Four years of college (BA, BS)	**−0.03 (−0.21 to 0.15)**		**0.22 (0.04 to 0.40)**		**0.1 (−0.07 to 0.27)**	
Graduate degree	**0.00 (Reference)**		**0.00 (Reference)**		**0.00 (Reference)**	
Annual household income						
≤$70,000	**0.27 (0.14 to 0.40)**	**<0.0001**	**0.27 (0.15 to 0.39)**	**<0.0001**	**0.40 (0.27 to 0.52)**	**<0.0001**
>$70,000	**0.00 (Reference)**		**0.00 (Reference)**		**0.00 (Reference)**	
No. of persons in household						
1–2	0.00 (Reference)	0.62	**0.00 (Reference)**	**0.06**	**0.00 (Reference)**	**<0.0001**
3–4	−0.09 (−0.22 to 0.03)		**0.07 (−0.06 to 0.20)**		**0.15 (0.02 to 0.28)**	
5–6	0.01 (−0.20 to 0.21)		**0.19 (−0.01 to 0.40)**		**0.57 (0.37 to 0.77)**	
7 or more	−0.02 (−0.42 to 0.38)		**0.17 (−0.23 to 0.57)**		**0.69 (0.29 to 1.09)**	
Receipt of public assistance						
Yes	0.09 (−0.03 to 0.21)	0.15	**0.39 (0.27 to 0.51)**	**<0.0001**	**0.57 (0.46 to 0.70)**	**<0.00001**
No	0.00 (Reference)		**0.00 (Reference)**		**0.00 (Reference)**	
**Biological characteristics**						
Prepregnancy BMI						
Underweight	0.17 (−0.18 to 0.51)	0.23	**0.09 (−0.26 to 0.43)**	**0.06**	**0.03 (−0.31 to 0.38)**	**0.001**
Normal weight	0.00 (Reference)		**0.00 (Reference)**		**0.00 (Reference)**	
Overweight	0.11 (−0.03 to 0.25)		**0.03 (−0.12 to 0.17)**		**0.13 (−0.01 to 0.27)**	
Obese	0.11 (−0.05 to 0.26)		**0.19 (0.03 to 0.34)**		**0.25 (0.10 to 0.41)**	
Parity (pregnancy index)						
1	**0.00 (Reference)**	**0.02**	0.00 (Reference)	0.30	0.00 (Reference)	0.50
2	**−0.45 (−0.76 to −0.14)**		−0.36 (−0.68 to −0.05)		0.05 (−0.27 to 0.37)	
3	**0.86 (−1.09 to 2.82)**		3.64 (1.70 to 5.59)		1.28 (−0.68 to 3.24)	
Gestational weight gain						
Inadequate	**0.19 (0.02 to 0.34)**	**0.02**	**0.14 (−0.03 to 0.30)**	**0.09**	**0.16 (0.001 to 0.32)**	**0.09**
Adequate	**0.00 (Reference)**		**0.00 (Reference)**		**0.00 (Reference)**	
Excessive	**0.20 (0.06 to 0.34)**		**−0.03 (−0.17 to 0.12)**		**0.14 (−0.003 to 0.28)**	
Gestational diabetes						
Yes	0.18 (−0.13 to 0.49)	0.26	−0.14 (−0.45 to 0.17)	0.39	0.04 (−0.27 to 0.35)	0.80
No			0.00 (Reference)		0.00 Reference	
Gestational age at delivery						
<37 weeks	0.13 (−0.11 to 0.38)	0.24	0.20 (−0.05 to 0.44)	0.12	−0.19 (−0.43 to 0.06)	0.13
≥37 weeks	0.00 (Reference)		0.00 (Reference)		0.00 (Reference)	
**Health behaviors**						
Smoked during pregnancy						
Did smoke	**0.24 (0.02 to 0.46)**	**0.03**	0.15 (−0.07 to 0.37)	0.18	**0.45 (0.23 to 0.66)**	**<0.0001**
Did not smoke	**0.00 (Reference)**		0.00 (Reference)		**0.00 (Reference)**	
HEI						
HEI >57	**0.00 (Reference)**	**0.007**	**0.00 (Reference)**	**0.0003**	**0.00 (Reference)**	**<0.0001**
HEI ≤57	**0.17 (0.05 to 0.29)**		**0.23 (0.10 to 0.35)**		**0.33 (0.21 to 0.45)**	
Physical activity						
≥150 min/week	0.07 (−0.05 to 0.19)	0.24	−0.10 (−0.22 to 0.02)	0.11	−0.09 (−0.21 to 0.03)	0.14
<150 min/week	0.00 (Reference)		0.00 (Reference)		0.00 (Reference)	

Bold text indicates characteristics that are statistically significantly associated with the respective Domain at alpha = 0.10.

Table 4 shows the multivariable results for perinatal correlates of each domain. Model 1 shows the perinatal correlates of Domain 1 (Feeling Overwhelmed). After mutual adjustment of variables associated with Domain 1 in unadjusted analyses, household income, parity, GWG, and HEI score remained significant. Lower household income (0.22 confidence interval [95% CI: 0.05–0.39]) in Domain 1 score for ≤$70,000 versus >$70,000, inadequate (0.22 [95% CI: 0.04–0.39]) or excessive (0.27 [95% CI: 0.11–0.42] for) GWG (vs. adequate GWG), and poorer diet quality (0.14 [95% CI: 0.00–0.28] for HEI ≤57 vs. >57) were each independently associated with higher scores for Domain 1. In addition, primiparas had a higher score for this domain (0.36 [95% CI: 0.02–0.71]) than multiparas.

**Table 4.** Multivariable Models of Maternal Sociodemographic and Perinatal Characteristics with Each Domain of Maternal Psychosocial Stress (*N* = 1,079)

	Model 1: Domain 1 (Feeling Overwhelmed)		Model 2: Domain 2 (Anhedonia)		Model 3: Domain 3 (Lack of Control)	
	Estimate (95% CI)	*p*	Estimate (95% CI)	*p*	Estimate (95% CI)	*p*
**Maternal sociodemographic characteristic**						
Maternal age	—	—	**0.02 (0.00 to 0.03)**	**0.02**	0.00 (−0.01 to 0.02)	0.77
Mother's race/ethnicity						
Hispanic	−0.09 (−0.27 to 0.10)	0.32	**0.19 (0.00 to 0.39)**	**0.04**	**−0.07 (−0.28 to 0.14)**	**0.02**
Non-Hispanic White	0.00 (Reference)		**0.00 (Reference)**		**0.00 (Reference)**	
Non-Hispanic Black	−0.02 (−0.24 to 0.20)		**0.14 (−0.12 to 0.39)**		**0.37 (0.10 to 0.63)**	
Other	0.20 (−0.08 to 0.47)		**−0.20 (−0.45 to 0.06)**		**0.12 (−0.14 to 0.39)**	
Mother's education						
Less than college graduate	0.01 (−0.17 to 0.19)	0.93	0.08 (−0.09 to 0.26)	0.36	0.16 (−0.02 to 0.35)	0.08
College graduate or higher	0.00 (Reference)		0.00 (Reference)		0.00 (Reference)	
Mother's birth country						
United States	—	—	0.00 (Reference)	0.61	0.00 (Reference)	0.12
Outside United States	—		0.05 (−0.15 to 0.26)		0.17 (−0.04 to 0.39)	
Mother's partner's birth country						
United States	—	—	0.00 (Reference)	0.21	0.00 (Reference)	0.08
Outside United States	—		0.12 (−0.07 to 0.31)		0.17 (−0.02 to 0.37)	
Household income						
≤$70,000	**0.22 (0.05 to 0.39)**	**0.01**	0.10 (−0.06 to 0.25)	0.23	0.03 (−0.13 to 0.20)	0.69
>$70,000	**0.00 (Reference)**		0.00 (Reference)		0.00 (Reference)	
No. of persons in household						
<5	—	—	0.00 (Reference)	0.83	0.00 (Reference)	0.08
≥5	—		−0.02 (−0.24 to 0.20)		0.21 (−0.02 to 0.44)	
Receipt of public assistance						
Yes	—	—	0.11 (−0.07 to 0.29)	0.23	**0.18 (−0.01 to 0.37)**	**0.06**
No	—		0.00 (Reference)		**0.00 (Reference)**	
**Biological characteristics**						
Prepregnancy BMI	—	—	0.00 (−0.02 to 0.01)	0.61	**0.00 (−0.01 to 0.02)**	0.41
Parity (at the time of index pregnancy)						
1	**0.36 (0.02 to 0.71)**	**0.04**	—	—	—	—
2–3	**0.00 (Reference)**		—		—	
Gestational weight gain						
Inadequate	**0.22 (0.04 to 0.39)**	**0.003**	—	—	—	—
Adequate	**0.00 (Reference)**		—		—	
Excessive	**0.27 (0.11 to 0.42)**		—		—	
**Health behaviors**						
Smoked during pregnancy						
Did smoke	0.11 (−0.15 to 0.36)	0.42	—	—	**0.32 (0.05 to 0.59)**	**0.02**
Did not smoke	0.00 (Reference)		—		**0.00 (Reference)**	
HEI						
HEI >57	**0.00 (Reference)**	**0.06**	**0.00 (Reference)**	**0.03**	0.00 (Reference)	0.53
HEI ≤57	**0.14 (0.00 to 0.28)**		**0.15 (0.02 to 0.28)**		0.04 (−0.09 to 0.18)	

All sociodemographic and perinatal characteristics with estimates provided in Table 4 were included in the multivariable models, respectively, as predictors.

Bold text indicates characteristics that are statistically significantly associated with the respective Domain at alpha = 0.10.

Model 2 in Table 4 shows the multivariable results for Domain 2 (Anhedonia). Here, older age (0.02 [95% CI: 0.00–0.03] per 1 year), Hispanic ethnicity (0.19 [95% CI: 0.00–0.39], and poor diet quality (0.15 [95% CI: 0.02–0.28]) were each independently associated with higher scores for this domain.

Model 3 in Table 4 shows the multivariable results for Domain 3 (Lack of Control). Non-Hispanic Black women had a higher score for this domain than non-Hispanic White women (0.37 [95% CI: 0.10–0.63]). Women with lower educational attainment (0.16 [95% CI: −0.02 to 0.35]), a partner born outside the United States (0.17 [95% CI: −0.02 to 0.37]), and households of ≥5 people (0.21 [95% CI: −0.02 to 0.44]), and those who received public assistance (0.18 [95% CI: −0.01 to 0.37]) and smoked during pregnancy (0.32 [95% CI: 0.05–0.59]) also had higher Domain 3 scores.

## Discussion

### Summary of main findings

In this analysis of 1,079 racially/ethnically and socioeconomically diverse pregnant women, we identified three domains of maternal stress based on EPDS and PSS responses. The first domain, which we named “Feeling Overwhelmed,” represented feelings of nervousness/stress, upset because of unexpected occurrences, and an inability to cope with responsibilities. The second domain, named “Anhedonia,” was driven by feeling unable to look forward to things with enjoyment or an inability to laugh at things. The third domain, named “Lack of Control,” was characterized by responses indicating a lack of confidence in handling personal problems, perceptions that things are not going one's way, and an inability to control irritations.

In multivariable analyses, correlates of Feeling Overwhelmed included biological traits during the perinatal period (primiparity, inadequate or excessive gestational weight gain), as well as health behavioral (poor diet quality) and sociodemographic (lower household income) characteristics. On the other hand, correlates of Anhedonia were primarily sociodemographic characteristics (older age and Hispanic ethnicity) and poor diet quality. Correlates of Lack of Control comprised several sociodemographic characteristics, including nonmodifiable traits/heritage (non-Hispanic Black race/ethnicity, non-United States nativity) and indicators of socioeconomic status (lower educational attainment, living with ≥5 persons in a household, and receipt of public assistance), and smoking during pregnancy. We discuss our findings below, including plausible biological and psychosocial pathways, acknowledging that this analysis was not designed to attribute causality to the correlates of each stress domain.

#### Domain 1: Feeling Overwhelmed

In unadjusted analysis, we identified several perinatal correlates of Feeling Overwhelmed, which were also associated with the other two stress domains. These variables included sociodemographic characteristics: non-White race/ethnicity, lower educational attainment, and lower household income; biological traits during the perinatal period: primiparity, and inadequate or excessive GWG; and health behaviors: poor diet quality and smoking during pregnancy. However, after mutual adjustment of these variables for one another, only one sociodemographic correlate (lower household income) remained significant, whereas all the biological traits (higher parity, and inadequate and excessive weight gain) and poor diet quality remained significant.

Because the sociodemographic characteristics are likely upstream of the biological and behavioral pathways, the attenuation of effects for the sociodemographic characteristics in the multivariable models suggests that they may be operating through biological and behavioral variables associated with feeling overwhelmed.

#### Domain 2: Anhedonia

In unadjusted analyses, all sociodemographic characteristics, and some biological (prepregnancy BMI, GWG) and behavioral characteristics (diet quality) were associated with Anhedonia. In multivariable analyses, only older age, Hispanic ethnicity, and poor diet quality remained significant. Given that age and ethnicity are nonmodifiable characteristics, the persistent association of these two variables with Anhedonia after accounting for downstream perinatal characteristics and health behaviors likely points toward the role of social experiences in influencing Anhedonia. This notion is supported by a recent study showing that experiences of racialized bias or discrimination affect emotional dysregulation.^36^ We also note some recent studies indicating a genetic contribution to anhedonia^37,38^ through this assessment were outside the scope of this analysis.

#### Domain 3: Lack of Control

Similar to Domain 2, all sociodemographic characteristics were associated with Lack of Control in unadjusted analyses, as were some biological (prepregnancy BMI, GWG) and behavioral traits (smoking during pregnancy and diet quality). In the multivariable model, several sociodemographic characteristics (non-Hispanic Black race/ethnicity, educational attainment, partner's nativity, household size, and receipt of public assistance) and one behavioral characteristic (smoking during pregnancy) remained significant.

The persistent associations of the sociodemographic characteristics with Lack of Control are not unexpected, and may represent correlated features of a specific group of individuals. For instance, non-Hispanic Black race/ethnicity, lower educational attainment, non-United States nativity, larger household size, and receipt of public assistance are shared characteristics of migrants in the Denver area.^39^ Recent migration is associated with uncertainty in personal and professional aspects of life,^40^ which may result in feeling a lack of control.

### Comparison to prior studies

Similar to Maxson et al.^17^ and Goldenberg et al.,^41^ we used individual responses from validated questionnaires to create latent constructs that capture distinct, multidimensional aspects of psychosocial stress. Maxson et al.^17^ used a group-based algorithm to categorize women into mutually exclusive stress domains, whereas Goldenberg et al. used factor analysis to identify components to create in a single abbreviated questionnaire from five distinct questionnaires that assess psychosocial status during pregnancy. We observe consistency in correlates of the psychosocial stress domains across studies. Maxson et al.^17^ found that women in the “vulnerable” stress profile group characterized by high depression and perceived stress, and low interpersonal support, paternal support, and self-efficacy, had higher odds of “risky health correlates” (*e.g.*, tobacco use and sexually transmitted infections) compared to those in the other groups.

We found a similar pattern with respect to health behaviors in our study. Specifically, women with poorer diet quality had higher factor scores for Feeling Overwhelmed and Anhedonia, and women who smoked during pregnancy had higher factor scores for Lack of Control. Characteristics indicating disadvantage, such as lower household income, or upstream interpersonal and social experiences based on one's race/ethnicity identity, were also associated with higher stress scores, although the associations were not consistent across all domains.^42^

Of note, the stress domains retained from PCA accounted for a relatively low percentage of variance in the EPDS and PSS questionnaire responses (∼11%), suggesting that there are other aspects of psychosocial stress that were not captured by these latent constructs. However, the percent variance accounted for by the individual domains (2.9% to 4.7%) is comparable to fifth (5%) and sixth (3.5%) factors retained in Goldenberg et al.'s study.^41^ It is also worth mentioning that, despite a general focus on latent constructs that account for a large amount of variance in the original dataset, the variance accounted for by a latent construct does not necessarily correlate with its relevance to an exposure or outcome. Prior studies have demonstrated that low variance factors (*i.e.*, 3%–4%, as in this analysis) were more relevant to a response variable than those that accounted for more variance in the dimension reduction step.^43^ This phenomenon transpires from the fact that the relationship of the latent construct(s) with an exposure or outcome is independent of variance accounted for in the high-dimensional dataset during dimension reduction.

### Strengths and limitations

Strengths of this study include the unsupervised data-driven approach to characterizing maternal stress during pregnancy, which allowed us to capture the complexity and multidimensional nature of psychosocial stress; a diverse study population recruited in a general risk setting, thereby enhancing generalizability; and rich covariate data, which allowed us to evaluate a number of sociodemographic, biological, and health behavioral characteristics during the perinatal period as correlates of stress domains that may serve as points of intervention for future studies.

This study also had some limitations. First, as mentioned above, the psychosocial stress domains identified in our study explained a relatively low percentage of interindividual variance in EPDS and PSS questionnaire responses. While the low variance does not discount the relevance of our findings, we acknowledge that there are likely other aspects of psychosocial stress that are not captured by the domains we retained in this analysis.

Second, we did not collect information on other important forms of stress, such as perceptions of racism and discrimination. Although we assessed race/ethnicity as a correlate of psychosocial stress, these are crude proxies and several racial/ethnic identities are not represented in our sample (*e.g.*, American Indian or Alaska Native and Asian). Therefore, we cannot comment on the factor scores in these groups in comparison to other groups.

Third, there is discourse surrounding the replicability of latent variables derived by PCA. We note, however, that studies reporting poor replicability of PCA factors used this procedure to reduce a large number of variables—for example, thousands of SNPs for derivation of ancestry principal components in genetic analyses.^44^ Such findings are countered by original research and systematic reviews demonstrating replicability of PCA-derived dietary patterns across populations in a homogenous cultural context.^45,46^ These studies used PCA to reduce a comparable number of variables to those of this study (*i.e.*, 20–35 variables) for construction of dietary patterns.^45,46^ Furthermore, we followed the PCA with an orthogonal rotation procedure, which has been shown to improve replicability.^47^ Future studies are required to assess the extent to which the stress domains identified herein are replicable in and generalizable to other populations.

Finally, the exploratory nature of this analysis and the relaxed threshold for statistical significance (alpha = 0.10) may be vulnerable to type 1 error. However, we emphasize that the data science task at hand was to explore and interpret associations as a way to inform future studies,^48,49^ as opposed to build a predictive model of maternal stress, the latter of which would suffer more from type 1 error.

## Conclusion

In a diverse population of pregnant women in Colorado, we identified Feeling Overwhelmed, Anhedonia, and Lack of Control as three distinct domains of psychosocial stress with differential associations to upstream sociodemographic, biological, and behavioral characteristics during the perinatal period. Our findings provide insight on patterns of maternal psychosocial stress during pregnancy that may have long-term implications for both the woman as well as her offspring. Moreover, the correlates of domains shed light on upstream determinants as well as possible biological and/or psychosocial pathways through which these experiences of stress manifest, thereby setting the stage for future studies to further explore these pathways and identify potential strategies for support or intervention.

Given the complexities of assessing psychosocial stress, future studies should consider mixed-method approaches that query women's lived experiences to inform interpretation of quantitative analyses such as the type performed herein. Examples include use of open-ended questions to better capture perceived stress, experiences of bias or discrimination, and other lived experiences of participants, followed by a quantitative analyses of such data^50^; or collection and use of qualitative data on stressors during pregnancy to inform development of stress management interventions.^51^ Such approaches create space for participants to provide cultural and contextual insight into their lived experiences, which lead not only to richer data but also more tailored and effective interventions.^52^

## Supplementary Material

Supplemental data

Supplemental data

## Abbreviations Used

BMI: body mass index
EPDS: Edinburgh Postnatal Depression Scale
GWG: gestational weight gain
HEI: Healthy Eating Index
PCA: principal component analysis
PPD: postpartum depression
PSS: Perceived Stress Scale

## References

[1] Cohen S, Janicki-Deverts D, Miller GE. Psychological stress and disease. JAMA 2007;298:1685–1687. 17925521 10.1001/jama.298.14.1685

[2] Entringer S, Buss C, Wadhwa PD. Prenatal stress and developmental programming of human health and disease risk: concepts and integration of empirical findings. Curr Opin Endocrinol Diabetes Obes 2010;17:507–516. 20962631 10.1097/MED.0b013e3283405921 PMC3124255

[3] Ronald A, Pennell CE, Whitehouse AJO. Prenatal maternal stress associated with ADHD and autistic traits in early childhood. Front Psychol 2011;1. [Epub ahead of print]; DOI: 10.3389/fpsyg.2010.00223. PMC3153828 21833278

[4] Zhu P, Hao JH, Tao RX, et al. Sex-specific and time-dependent effects of prenatal stress on the early behavioral symptoms of ADHD: A longitudinal study in China. Eur Child Adolesc Psychiatry 2015;24:1139–1147. 25791080 10.1007/s00787-015-0701-9

[5] Dorrington S, Zammit S, Asher L, Evans J, Heron J, Lewis G. Perinatal maternal life events and psychotic experiences in children at twelve years in a birth cohort study. Schizophr Res 2014;152:158–163. 24275580 10.1016/j.schres.2013.11.006 PMC3906533

[6] Class QA, Abel KM, Khashan AS, et al. Offspring psychopathology following preconception, prenatal and postnatal maternal bereavement stress. Psychol Med 2014;44:71–84. 23591021 10.1017/S0033291713000780 PMC3766407

[7] Grizenko N, Fortier ME, Zadorozny C, et al. Maternal stress during pregnancy, ADHD symptomatology in children and genotype: Gene-environment interaction. J Can Acad Child Adolesc Psychiatry 2012;21:9–15. 22299010 PMC3269259

[8] Van den Bergh BRH, van den Heuvel MI, Lahti M, et al. Prenatal developmental origins of behavior and mental health: The influence of maternal stress in pregnancy. Neurosci Biobehav Rev 2017:S0149763416307345. 10.1016/j.neubiorev.2017.07.003 28757456

[9] Robinson M, Mattes E, Oddy WH, et al. Prenatal stress and risk of behavioral morbidity from age 2 to 14 years: The influence of the number, type, and timing of stressful life events. Dev Psychopathol 2011;23:507–520. 23786692 10.1017/S0954579411000241

[10] Tearne JE, Allen KL, Herbison CE, et al. The association between prenatal environment and children's mental health trajectories from 2 to 14 years. Eur Child Adolesc Psychiatry 2015;24:1015–1024. 25431038 10.1007/s00787-014-0651-7

[11] Li J, Olsen J, Vestergaard M, Obel C. Attention-deficit/hyperactivity disorder in the offspring following prenatal maternal bereavement: A nationwide follow-up study in Denmark. Eur Child Adolesc Psychiatry 2010;19:747–753. 20495989 10.1007/s00787-010-0113-9

[12] Sable MR, Wilkinson DS. Impact of perceived stress, major life events and pregnancy attitudes on low birth weight. Fam Plann Perspect 2000;32:288. 11138865

[13] Weber KA, Carmichael SL, Yang W, Tinker SC, Shaw GM; National Birth Defects Prevention Study. Periconceptional stressors and social support and risk for adverse birth outcomes. BMC Pregnancy Childbirth 2020;20:487. 32831042 10.1186/s12884-020-03182-6 PMC7446063

[14] Van den Bergh BRH, Mulder EJH, Mennes M, Glover V. Antenatal maternal anxiety and stress and the neurobehavioural development of the fetus and child: Links and possible mechanisms. A review. Neurosci Biobehav Rev 2005;29:237–258. 15811496 10.1016/j.neubiorev.2004.10.007

[15] Choi B, Shim G, Jeong B, Jo S. Data-driven analysis using multiple self-report questionnaires to identify college students at high risk of depressive disorder. Sci Rep 2020;10:7867. 32398788 10.1038/s41598-020-64709-7 PMC7217968

[16] Niemann U, Brueggemann P, Boecking B, et al. Phenotyping chronic tinnitus patients using self-report questionnaire data: Cluster analysis and visual comparison. Sci Rep 2020;10:16411. 33009468 10.1038/s41598-020-73402-8 PMC7532444

[17] Maxson PJ, Edwards SE, Valentiner EM, Miranda ML. A multidimensional approach to characterizing psychosocial health during pregnancy. Matern Child Health J 2016;20:1103–1113. 27107859 10.1007/s10995-015-1872-1

[18] Dunteman G. Principal components analysis. SAGE Publications, Inc., 1989. [Epub ahead of print]; DOI: 10.4135/9781412985475.

[19] Sauder KA, Starling AP, Shapiro AL, et al. Diet, physical activity and mental health status are associated with dysglycaemia in pregnancy: The Healthy Start Study. Diabet Med 2016;33:663–667. 26872289 10.1111/dme.13093 PMC4883104

[20] Cohen S, Kamarck T, Mermelstein R. A global measure of perceived stress. J Health Soc Behav 1983;24:385–396. 6668417

[21] Cox JL, Holden JM, Sagovsky R. Detection of postnatal depression: Development of the 10-item Edinburgh Postnatal Depression Scale. Br J Psychiatry 1987;150:782–786. 3651732 10.1192/bjp.150.6.782

[22] Lavoie JAA, Douglas KS. The Perceived Stress Scale: Evaluating configural, metric and scalar invariance across mental health status and gender. J Psychopathol Behav Assess 2012;34:48–57.

[23] Khanlari S, Barnett Am B, Ogbo FA, Eastwood J. Re-examination of perinatal mental health policy frameworks for women signalling distress on the Edinburgh Postnatal Depression Scale (EPDS) completed during their antenatal booking-in consultation: A call for population health intervention. BMC Pregnancy Childbirth 2019;19:221. 31266468 10.1186/s12884-019-2378-4 PMC6604146

[24] Combs MA, Canu WH, Broman-Fulks JJ, Rocheleau CA, Nieman DC. Perceived stress and ADHD symptoms in adults. J Atten Disord 2015;19:425–434. 23034340 10.1177/1087054712459558

[25] Nast I, Bolten M, Meinlschmidt G, Hellhammer DH. How to measure prenatal stress? A systematic review of psychometric instruments to assess psychosocial stress during pregnancy: Assessment of psychosocial stress during pregnancy. Paediatr Perinat Epidemiol 2013;27:313–322. 23772932 10.1111/ppe.12051

[26] Perng W, Francis EC, Schuldt C, Barbosa G, Dabelea D, Sauder KA. Pre- and perinatal correlates of ideal cardiovascular health during early childhood: A prospective analysis in the Healthy Start Study. J Pediatr 2021;234:187–194. 33741366 10.1016/j.jpeds.2021.03.014 PMC8238850

[27] Starling AP, Brinton JT, Glueck DH, et al. Associations of maternal BMI and gestational weight gain with neonatal adiposity in the Healthy Start study. Am J Clin Nutr 2015;101:302–309. 25646327 10.3945/ajcn.114.094946 PMC4307203

[28] National Heart, Lung, and Blood Institute (NHLBI) Obesity Education Initiative Expert Panel on the Identification, Evaluation, and Treatment of Obesity in Adults (US). Clinical guidelines of the identification, evaluation, and treatment of overweight and obesity in adults: The evidence report. Bethesda, MD: National Heart, Lung, and Blood Institute, 1998.

[29] Institute of Medicine. Weight gain during pregnancy: Reexamining the guidelines. Washington, DC: The National Academics Press, 2009. 20669500

[30] Raghavan S, Zhang W, Yang IV, et al. Association between gestational diabetes mellitus exposure and childhood adiposity is not substantially explained by offspring genetic risk of obesity. Diabet Med 2017;34:1696–1700. 29048747 10.1111/dme.13529 PMC6880873

[31] Shapiro ALB, Kaar JL, Crume TL, et al. Maternal diet quality in pregnancy and neonatal adiposity: The Healthy Start Study. Int J Obes (Lond) 2016;40:1056–1062. 27133623 10.1038/ijo.2016.79 PMC5356926

[32] Francis EC, Dabelea D, Shankar K, Perng W. Maternal diet quality during pregnancy is associated with biomarkers of metabolic risk among male offspring. Diabetologia 2021. [Epub ahead of print]; DOI: 10.1007/s00125-021-05533-0. PMC8499858 34370046

[33] Chasan-Taber L, Schmidt MD, Roberts DE, Hosmer D, Markenson G, Freedson PS. Development and validation of a Pregnancy Physical Activity Questionnaire. Med Sci Sports Exerc 2004;36:1750–1760. 15595297 10.1249/01.mss.0000142303.49306.0d

[34] Harrod CS, Chasan-Taber L, Reynolds RM, et al. Physical activity in pregnancy and neonatal body composition: The Healthy Start study. Obstet Gynecol 2014;124(2 Pt 1):257–264. 25004346 10.1097/AOG.0000000000000373 PMC4111108

[35] How Much Physical Activity Do Adults Need? Centers for Disease Control and Prevention, 2020. Available at: https://www.cdc.gov/physicalactivity/basics/adults/index.htm#:~:text=Each%20week%20adults%20need%20150,Physical%20Activity%20Guidelines%20for%20Americans

[36] Mekawi Y, Watson-Singleton NN, Kuzyk E, et al. Racial discrimination and posttraumatic stress: Examining emotion dysregulation as a mediator in an African American community sample. Eur J Psychotraumatol 2020;11:1824398. 33244363 10.1080/20008198.2020.1824398 PMC7678677

[37] Ward J, Lyall LM, Bethlehem RAI, et al. Novel genome-wide associations for anhedonia, genetic correlation with psychiatric disorders, and polygenic association with brain structure. Transl Psychiatry 2019;9:327. 31797917 10.1038/s41398-019-0635-y PMC6892870

[38] Ren H, Fabbri C, Uher R, et al. Genes associated with anhedonia: A new analysis in a large clinical trial (GENDEP). Transl Psychiatry 2018;8:150. 30104601 10.1038/s41398-018-0198-3 PMC6089928

[39] Migration Policy Institute. Colorado State Immigration Data Profiles. 2020. Available at: https://www.migrationpolicy.org/data/state-profiles/state/demographics/CO Last accessed December 10, 2021.

[40] Daftary AH. Living with uncertainty: Perceptions of well-being among Latinx young adults in immigrant family systems. Fam Relat 2020;69:51–62.

[41] Goldenberg RL, Hickey CA, Cliver SP, Gotlieb S, Woolley TW, Hoffman HJ. Abbreviated scale for the assessment of psychosocial status in pregnancy: Development and evaluation. Acta Obstet Gynecol Scand Suppl 1997;165:19–29. 9219452

[42] Adkins-Jackson PB, Chantarat T, Bailey ZD, Ponce NA. Measuring Structural Racism: A guide for epidemiologists and other health researchers. Am J Epidemiol 2021:kwab239. [Epub ahead of print]; DOI: 10.1093/aje/kwab239. PMC9077112 34564723

[43] Jolliffe IT. A note on the use of principal components in regression. Appl Stat 1982;31:300.

[44] Elhaik E. Why most principal component analyses (PCA) in population genetic studies are wrong. Genetics 2021. [Epub ahead of print]; DOI: 10.1101/2021.04.11.439381. PMC9424212 36038559

[45] Castelló A, Lope V, Vioque J, et al. Reproducibility of data-driven dietary patterns in two groups of adult Spanish women from different studies. Br J Nutr 2016;116:734–742. 27374250 10.1017/S000711451600252X

[46] Murakami K, Shinozaki N, Fujiwara A, et al. A systematic review of principal component analysis-derived dietary patterns in Japanese adults: Are major dietary patterns reproducible within a country? Adv Nutr 2019;10:237–249. 30785205 10.1093/advances/nmy079 PMC6416039

[47] Rennie KM. Rennie, Kimberly M.. “Exploratory and Confirmatory Rotation Strategies in Exploratory Factor Analysis.” (1997). 1997. Available at: https://www.semanticscholar.org/paper/Exploratory-and-Confirmatory-Rotation-Strategies-in-Rennie/20010bbc89218313c8f8396694b87c5a2da429ed Last accessed January 17, 2022.

[48] Laubach ZM, Murray EJ, Hoke KL, Safran RJ, Perng W. A biologist's guide to model selection and causal inference. Proc R Soc B 2021;288:20202815. 10.1098/rspb.2020.2815 PMC7893255 33499782

[49] Conroy S, Murray EJ. Let the question determine the methods: Descriptive epidemiology done right. Br J Cancer 2020;123:1351–1352. 32814836 10.1038/s41416-020-1019-z PMC7592039

[50] Frazier T, Hogue CJ, Yount KM. The development of the healthy pregnancy stress scale, and validation in a sample of low-income African American women. Matern Child Health J 2018;22:247–254. 29190008 10.1007/s10995-017-2396-7 PMC5808873

[51] Bloom T, Glass N, Curry MA, Hernandez R, Houck G. Maternal stress exposures, reactions, and priorities for stress reduction among lowincome, urban women. J Midwifery Womens Health 2013;58:167–174. 23278984 10.1111/j.1542-2011.2012.00197.x PMC4317357

[52] Sullivan-Bolyai S, Bova C, Harper D. Developing and refining interventions in persons with health disparities: The use of qualitative description. Nurs Outlook 2005;53:127–133. 15988449 10.1016/j.outlook.2005.03.005

