# Supplementary material for: Characterization of Maternal Psychosocial Stress During Pregnancy: The Healthy Start Study
Source: Womens Health Rep (New Rochelle). 2022 Aug 4;3(1):698–708. doi: 10.1089/whr.2022.0011 (PMC9436384; doi:10.1089/whr.2022.0011)
Supplement: Supplemental data [file Suppl_TableS1.docx]

**Supplemental Tables**

| **Table S1: Maternal EPDS and PSS Questions, Healthy Start** |  |  |
| --- | --- | --- |
|  | **N** | **%** |
| **Perceived Stress Scale Questions** |  |  |
| 1. In the last month, how often have you been upset about something that happened unexpectedly? (0=never, 1=almost never, 2=sometimes, 3=fairly often, 4=very often) |  |  |
| Never | 215 | 19.9 |
| Almost never | 410 | 38 |
| Sometimes | 366 | 33.9 |
| Fairly often | 71 | 6.6 |
| Very often | 17 | 1.6 |
| 2. In the last month, how often have you felt that you were unable to control important things in your life? (0=never, 1=almost never, 2=sometimes, 3=fairly often, 4=very often) |  |  |
| Never | 439 | 40.7 |
| Almost never | 344 | 31.9 |
| Sometimes | 199 | 18.4 |
| Fairly often | 69 | 6.4 |
| Very often | 28 | 2.6 |
| 3. In the last month, how often how often have you felt nervous and stressed? (0=never, 1=almost never, 2=sometimes, 3=fairly often, 4=very often) |  |  |
| Never | 116 | 10.8 |
| Almost never | 296 | 27.4 |
| Sometimes | 488 | 45.2 |
| Fairly often | 134 | 12.4 |
| Very often | 45 | 4.2 |
| 4. In the last month, how often have you felt confident about your ability to handle your personal problems? (4=never, 3=almost never, 2=sometimes, 1=fairly often, 0=very often) |  |  |
| Very often | 505 | 46.8 |
| Fairly often | 352 | 32.6 |
| Sometimes | 155 | 14.4 |
| Almost never | 40 | 3.7 |
| Never | 27 | 2.5 |
| 5. In the last month, how often have you felt that things were going your way? (4=never, 3=almost never, 2=sometimes, 1=fairly often, 0=very often) |  |  |
| Very often | 277 | 25.7 |
| Fairly often | 484 | 44.9 |
| Sometimes | 234 | 21.7 |
| Almost never | 63 | 5.8 |
| Never | 21 | 2 |
| 6. In the last month, how often have you found that you could not cope with all the things you had to do? (0=never, 1=almost never, 2=sometimes, 3=fairly often, 4=very often) |  |  |
| Never | 352 | 32.6 |
| Almost never | 411 | 38.1 |
| Sometimes | 245 | 22.7 |
| Fairly often | 49 | 4.5 |
| Very often | 22 | 2 |
| 7. In the past month, how often have you been able to control irritations in your life? (4=never, 3=almost never, 2=sometimes, 1=fairly often, 0=very often) |  |  |
| Very often | 278 | 25.8 |
| Fairly often | 448 | 41.5 |
| Sometimes | 271 | 25.1 |
| Almost never | 58 | 5.4 |
| Never | 24 | 2.2 |
| 8. In the past month, how often have you felt that you were on top of things? (4=never, 3=almost never, 2=sometimes, 1=fairly often, 0=very often) |  |  |
| Very often | 263 | 24.4 |
| Fairly often | 509 | 47.2 |
| Sometimes | 239 | 22.2 |
| Almost never | 57 | 5.3 |
| Never | 11 | 1 |
| 9. In the past month, how often have you been angered because of things that were outside of your control? (0=never, 1=almost never, 2=sometimes, 3=fairly often, 4=very often) |  |  |
| Never | 226 | 21 |
| Almost never | 429 | 39.8 |
| Sometimes | 299 | 27.7 |
| Fairly often | 102 | 9.5 |
| Very often | 23 | 2.1 |
| 10. In the past month, how often have you felt difficulties were piling up so high that you could not overcome them? (0=never, 1=almost never, 2=sometimes, 3=fairly often, 4=very often) |  |  |
| Never | 473 | 43.8 |
| Almost never | 390 | 36.1 |
| Sometimes | 167 | 15.5 |
| Fairly often | 38 | 3.5 |
| Very often | 11 | 1 |
| **Edinburgh Postnatal Depression Scale Questions** |  |  |
| 1. In the past seven days, I have been able to laugh and see the funny side of things. (0=As much as I always could, 1=Not quite so much now, 2=Definitely not so much now, 3=Not at all) |  |  |
| As much as I always could | 964 | 89.3 |
| Not quite so much now | 106 | 9.8 |
| Definitely not so much now | 6 | 0.4 |
| Not at all | 3 | 0.3 |
| 2. I have looked forward with enjoyment to things. (0=As much as I always could, 1=Not quite so much now, 2=Definitely not so much now, 3=Not at all) |  |  |
| As much as I always could | 922 | 85.5 |
| Not quite so much now | 143 | 13.3 |
| Definitely not so much now | 12 | 1.1 |
| Not at all | 2 | 0.2 |
| 3. I have blamed myself unnecessarily when things went wrong. (3=As much as I always could, 2=Not quite so much now, 1=Definitely not so much now, 0=Not at all) |  |  |
| Not at all | 31 | 2.9 |
| Definitely not so much now | 216 | 20 |
| Not quite so much now | 483 | 44.8 |
| As much as I always could | 349 | 32.3 |
| 4. I have been anxious or worried for no good reason. (3=As much as I always could, 2=Not quite so much now, 1=Definitely not so much now, 0=Not at all) |  |  |
| Not at all | 29 | 2.7 |
| Definitely not so much now | 313 | 29 |
| Not quite so much now | 358 | 33.2 |
| As much as I always could | 379 | 35.1 |
| 5. I have felt scared or panicky for no very good reason. (3=As much as I always could, 2=Not quite so much now, 1=Definitely not so much now, 0=Not at all) |  |  |
| Not at all | 12 | 1.1 |
| Definitely not so much now | 132 | 12.2 |
| Not quite so much now | 270 | 25 |
| As much as I always could | 665 | 61.6 |
| 6. Things have been getting on top of me. (3=As much as I always could, 2=Not quite so much now, 1=Definitely not so much now, 0=Not at all) |  |  |
| Not at all | 12 | 1.1 |
| Definitely not so much now | 167 | 15.5 |
| Not quite so much now | 453 | 42 |
| As much as I always could | 447 | 41.4 |
| 7. I have been so unhappy that I have difficulty sleeping. (3=As much as I always could, 2=Not quite so much now, 1=Definitely not so much now, 0=Not at all) |  |  |
| Not at all | 12 | 1.1 |
| Definitely not so much now | 75 | 7 |
| Not quite so much now | 193 | 17.9 |
| As much as I always could | 799 | 74.1 |
| 8. I have felt sad or miserable. (3=As much as I always could, 2=Not quite so much now, 1=Definitely not so much now, 0=Not at all) |  |  |
| Not at all | 10 | 0.9 |
| Definitely not so much now | 67 | 6.2 |
| Not quite so much now | 371 | 34.4 |
| As much as I always could | 631 | 58.5 |
| 9. I have been so unhappy that I have been crying. (3=As much as I always could, 2=Not quite so much now, 1=Definitely not so much now, 0=Not at all) |  |  |
| Not at all | 7 | 0.7 |
| Definitely not so much now | 44 | 4.1 |
| Not quite so much now | 383 | 35.5 |
| As much as I always could | 645 | 59.8 |
| 10. The thought of harming myself has occurred to me. (3=As much as I always could, 2=Not quite so much now, 1=Definitely not so much now, 0=Not at all) |  |  |
| Not at all | 0 | 0 |
| Definitely not so much now | 7 | 0.7 |
| Not quite so much now | 29 | 2.7 |
| As much as I always could | 1043 | 96.7 |
| ^a^ Frequencies and percentages may not add up to the total sample size due to missing values. | | |
