# Supplementary material for: Characterization of Maternal Psychosocial Stress During Pregnancy: The Healthy Start Study
Source: Womens Health Rep (New Rochelle). 2022 Aug 4;3(1):698–708. doi: 10.1089/whr.2022.0011 (PMC9436384; doi:10.1089/whr.2022.0011)
Supplement: Supplemental data [file Suppl_TableS2.docx]

**Supplemental Tables**

| **Table S2: Pearson's Correlation Coefficients across demographic and perinatal characteristics** | | | | | | | | | | | | | | | | |
| --- | --- | --- | --- | --- | --- | --- | --- | --- | --- | --- | --- | --- | --- | --- | --- | --- |
|  | MA | AHH | CHH | PPBMI | Parity | HEI | PA | GA | MBC | PBC | ME | HHI | PAT | GA | GDM | GS |
| Maternal age (MA) | 1.00 | -0.06 | 0.03 | 0.03 | 0.04 | 0.37 | -0.03 | 0.08 | 0.04 | 0.01 | 0.62 | 0.53 | -0.43 | 0.02 | 0.04 | -0.14 |
| Adults in household (AHH) |  | 1.00 | 0.07 | -0.07 | -0.05 | 0.02 | 0.01 | 0.06 | -0.02 | 0.08 | -0.05 | 0.29 | -0.01 | 0.05 | -0.02 | 0.03 |
| Children in household (CHH) |  |  | 1.00 | 0.14 | 0.13 | -0.14 | 0.08 | -0.03 | 0.10 | 0.17 | -0.26 | -0.19 | 0.25 | -0.08 | 0.00 | 0.11 |
| Pre-pregnancy BMI (PPBMI) |  |  |  | 1.00 | -0.03 | -0.15 | -0.03 | -0.06 | 0.07 | 0.15 | -0.18 | -0.24 | 0.18 | 0.02 | 0.11 | 0.01 |
| Parity |  |  |  |  | 1.00 | 0.00 | 0.02 | 0.00 | -0.07 | -0.06 | 0.03 | -0.01 | 0.00 | -0.07 | -0.01 | 0.04 |
| HEI |  |  |  |  |  | 1.00 | -0.10 | 0.06 | 0.04 | 0.03 | 0.41 | 0.39 | -0.29 | -0.02 | 0.00 | -0.26 |
| Physical Activity (PA) |  |  |  |  |  |  | 1.00 | 0.02 | -0.15 | -0.11 | -0.01 | -0.07 | 0.07 | -0.02 | -0.04 | 0.07 |
| Gestational age (GA) |  |  |  |  |  |  |  | 1.00 | -0.01 | -0.04 | 0.07 | 0.13 | -0.10 | 0.18 | -0.09 | -0.04 |
| Mother's birth country (MBC) |  |  |  |  |  |  |  |  | 1.00 | 0.47 | -0.18 | -0.10 | 0.11 | -0.10 | 0.00 | -0.12 |
| Partner's birth country (PBC) |  |  |  |  |  |  |  |  |  | 1.00 | -0.22 | -0.17 | 0.18 | -0.09 | 0.00 | -0.09 |
| Maternal education (ME) |  |  |  |  |  |  |  |  |  |  | 1.00 | 0.68 | -0.58 | 0.04 | 0.01 | -0.20 |
| Household income (HHI) |  |  |  |  |  |  |  |  |  |  |  | 1.00 | -0.64 | 0.05 | -0.03 | -0.27 |
| Public assistance (PAT) |  |  |  |  |  |  |  |  |  |  |  |  | 1.00 | -0.04 | 0.02 | 0.19 |
| Gestational weight gain (GWG) |  |  |  |  |  |  |  |  |  |  |  |  |  | 1.00 | -0.06 | -0.06 |
| Gestational diabetes (GDM) |  |  |  |  |  |  |  |  |  |  |  |  |  |  | 1.00 | -0.02 |
| Gestational smoking (GS) |  |  |  |  |  |  |  |  |  |  |  |  |  |  |  | 1.00 |
